# Geometry-Dependent Variability in Rotational Rheometry of Whole Blood: Implications for Flow Curve Measurement and Protocol Standardization

**DOI:** 10.3390/mps9050150

**Published:** 2026-10-14

**Authors:** Nebojša Pavlović, Biljana Vučković, Dušan Sedlarević, Nebojša Kladar, Stana Tokić, Ana Stupin, Krešimir Šolić, Veljko Krstonošić

**Affiliations:** 1 Department of Pharmacy, Faculty of Medicine, University of Novi Sad, 21000 Novi Sad, Serbia; nebojsa.kladar@mf.uns.ac.rs (N.K.); veljko.krstonosic@mf.uns.ac.rs (V.K.); 2 Department of Pathophysiology and Laboratory Medicine, Faculty of Medicine, University of Novi Sad, 21000 Novi Sad, Serbia; biljana.vuckovic@mf.uns.ac.rs (B.V.); dusan.sedlarevic@mf.uns.ac.rs (D.S.); 3 University Clinical Center of Vojvodina, Service for Supply of Blood and Blood Products—Hospital Blood Bank, 21000 Novi Sad, Serbia; 4 Department of Laboratory Medicine and Pharmacy, Faculty of Medicine, Josip Juraj Strossmayer University of Osijek, 31000 Osijek, Croatia; stokic@mefos.hr; 5 Department of Physiology and Immunology, Faculty of Medicine, Josip Juraj Strossmayer University of Osijek, 31000 Osijek, Croatia; astupin@mefos.hr; 6 Department of Medical Statistics and Medical Informatics, Faculty of Medicine, Josip Juraj Strossmayer University of Osijek, 31000 Osijek, Croatia; kresimir@mefos.hr

**Keywords:** blood rheology, flow curve, geometry, double gap, consistency index

## Abstract

Hemorheology has clear physiological relevance and potential diagnostic value, emphasizing the need for reliable blood rheological measurements. Rotational rheometry is widely used for the characterization of blood flow behavior, yet the influence of measuring geometry on rheological parameters remains insufficiently investigated and standardized. This study evaluated geometry-dependent variability in whole blood rheometry by comparing a double-gap coaxial cylinder (CC27 DG) with two cone–plate geometries differing in size and cone angle (C50 1° and C35 2°). Blood samples from five healthy volunteers were analyzed using an identical shear protocol from 0 to 100 s^−1^, and flow curves were fitted using the Power law model. All geometries consistently demonstrated non-Newtonian, shear-thinning behavior. Significant overall geometry effects were observed for the consistency index *K* (*p* = 0.022) and Power law goodness-of-fit *r* (*p* = 0.015), whereas no significant overall effect was detected for *n* (*p* = 0.247). While no pairwise comparison reached statistical significance after Holm adjustment, CC27 DG showed the lowest mean *K*, the highest mean *r*, and the smoothest flow curves, and C50 1° produced smooth curves with a reduced sample volume requirement. These findings support explicit reporting of measuring geometry and its consideration in the standardization of hemorheological protocols.

## 1. Introduction

Hemorheology studies the flow and deformation of blood and interactions among its cellular components and the vascular system [1]. Blood rheological properties influence vascular resistance, tissue perfusion, and microcirculation. Altered blood and plasma viscosity have been associated with cardiovascular and metabolic disorders, supporting interest in hemorheological measurements as research tools and potential markers of cardiovascular dysfunction [2,3,4].

Blood is a concentrated heterogeneous suspension of red blood cells (RBCs or erythrocytes), white blood cells (WBCs or leukocytes) and platelets (thrombocytes) in plasma as an aqueous polymeric and ionic solution [1]. The erythrocyte volume fraction (hematocrit) is typically 40–45% and represents the major determinant of whole blood viscosity. However, blood rheology is also governed by plasma viscosity and composition, as well as erythrocyte deformability and aggregation. At low shear rates, erythrocytes form three-dimensional aggregates known as rouleaux, leading to the increased blood viscosity, whereas increasing shear rate leads to the aggregate disruption and deformation. Therefore, whole blood exhibits shear-thinning rheological behavior together with more complex rheological characteristics, including viscoelasticity and thixotropy. Plasma, on the other hand, is usually approximated as a Newtonian fluid, although weak viscoelastic behavior has also been reported under specific experimental conditions [5,6]. Due to this complex behavior, various mathematical models have been proposed to describe blood flow properties, including Newtonian, Power law, Casson, Carreau, Carreau–Yasuda, Quemada, and other generalized Newtonian or viscoelastic models. The suitability of a given model depends on the investigated shear-rate range, the experimental protocol, and the intended application [7].

Several experimental techniques have been developed for investigating different aspects of blood rheology. Conventional rotational, steady-shear rheometry is commonly used to determine bulk flow behavior, whereas oscillatory rheological measurements can provide information on viscoelastic and time-dependent properties. Other approaches include capillary viscometry, microfluidic methods, microscopy- or optical-based aggregation measurements, and ektacytometry for dynamic assessment of erythrocyte deformability [6]. These techniques are complementary to the conventional ones, because they assess different structural and flow properties of blood. However, rotational rheometry remains one of the most widely used hemorheological methods. Considering the physiological and potential diagnostic relevance of blood rheology, along with the influence of both preanalytical and analytical conditions on the results, the widespread use of rotational rheometry further emphasizes the need for standardized measurement protocols.

In rotational rheometry, the selection of appropriate geometry of rotational elements is essential, as it has a critical role in determining rheological parameters. In addition, the selection of measurement geometry must balance precision with practical considerations, such as ease of use or cost. Conventional geometries such as coaxial cylinder or cone–plate systems provide well-defined shear rates and are generally preferred for quantitative rheological characterization, whereas alternative or irregular geometries, such as helical ribbons, anchors and paddles, are frequently used to overcome limitations associated with materials displaying complex characteristics, including wall slip or particle settling. Differences in measurement geometry may introduce systematic variations in the measured rheological response, particularly for non-Newtonian and particulate systems [8].

The impact of measurement geometry has been demonstrated for a variety of complex fluids and semisolids. For example, concentric cylinder geometries were shown to provide more consistent flow data and more reliable estimation of Power law parameters than cone–plate or parallel-plate systems for hazelnut pastes [9], whereas the calculated consistency index (*K*) and flow behavior index (*n*) values for coarse particulate food suspensions (model food suspensions consisting of seeds suspended in a shear-thinning fluid designed to mimic Mexican sauces) depended on the selected measuring geometry and applied shear rate range, with the ribbon geometry being the most representative under those experimental conditions [10]. Similarly, studies on particle-rich debris flow slurries have demonstrated that measurement geometry (concentric cylinder, parallel plate, vane, ball) and wall slip can substantially affect the fitted rheological parameters, particularly at low shear rates [11]. Furthermore, an interlaboratory comparison of cement paste rheological properties showed that measurements carried out using different rheometers and measuring geometries are not directly interchangeable without adequate corrections, emphasizing the importance of measurement geometry for obtaining comparable rheological results [12].

Despite the existing evidence in various complex fluids, the influence of rotational element geometry on the rheological characterization of whole blood has not yet been fully elucidated and the clear knowledge gap still remains regarding the effect of rotational rheometer geometry on flow curve determination, fitted rheological parameters, and model quality when blood samples are analyzed under the same experimental conditions. Therefore, the aim of the present study was to determine the influence of measurement geometry on the rheological characterization of whole blood by comparing a double gap coaxial cylinder geometry with two cone–plate geometries differing in cone diameter and cone angle. The analytical performance of the measurement geometries was assessed based on the calculated values of the consistency index (*K*) and flow behavior index (*n*). In addition, the geometries were evaluated with respect to the quality of the Power law model fit, as well as practical considerations related to sample volume requirements.

## 2. Materials and Methods

### 2.1. Blood Samples and Ethical Approval

Whole blood samples were collected from five healthy adult volunteers recruited at the Blood Transfusion Institute of Vojvodina, Novi Sad, Serbia. The study population consisted of three male and two female donors, aged 20–43 years, with hematocrit values in a narrow range from 0.41 to 0.44. All participants provided written informed consent prior to blood collection. The study protocol was approved by the Ethics Committee of the Faculty of Medicine, University of Novi Sad (No. 01-39/257/1) and the Ethics Committee of the Blood Transfusion Institute of Vojvodina (No. 01-10/24).

Blood was collected into tubes with EDTA as an anticoagulant, and the samples were kept at controlled ambient temperature (20–22 °C) until analysis. All rheological measurements were performed within two hours of blood collection (samples from one or two donors were analyzed per day), and samples were gently mixed before testing to minimize the effects of storage time and temperature on blood rheological properties [13]. Measurements did not follow a fixed geometry sequence. Since each flow-curve measurement lasted 120 s, the measurements with different geometries were performed within a time window of less than 30 min for each donor.

### 2.2. Rheological Measurements

Rheological blood tests were conducted using a rotational rheometer (HAAKE MARS, Thermo Fisher Scientific, Karlsruhe, Germany) equipped with a Peltier temperature control system. Measurements were carried out at a controlled temperature of 25.0 ± 0.1 °C using three measuring geometries: a double-gap coaxial cylinder (CC27 DG), a 50 mm diameter cone–plate geometry with a 1° cone angle (C50 1°), and a 35 mm diameter cone–plate geometry with a 2° cone angle (C35 2°). A temperature of 25 °C was used as a controlled laboratory reference condition for comparing geometries; the study was not designed to determine physiological blood-viscosity reference values at 37 °C. The approximate sample volumes required for the measurements were 3.0 mL, 1.0 mL, and 0.4 mL for the CC27 DG, C50 1°, and C35 2° geometries, respectively. The individual geometry characteristics (dimensions and factory conversion factors) are provided in Table S1. RheoWin 4 Data Manager software (v. 4.91.0011) calculated shear stress and shear rate from torque and angular velocity using τ = *AT* and $\dot{\gamma }$ = *M*ω. For CC27 DG, no additional non-Newtonian correction was applied, and no fitted or assumed flow index was used in these conversions.

For each donor, two separate measurements were performed using each of the three geometries under the same experimental conditions. Flow curves were recorded using a stepwise linear shear-rate program. They were recorded by increasing the shear rate from 0 to 100 s^−1^ over 50 s, followed by a 20 s period at a constant shear rate of 100 s^−1^. Eventually, the shear rate was decreased from 100 to 0 s^−1^ over an additional 50 s. The nominal interval between consecutive data points was 1 s. Differences between the ascending and descending branches were small over most of the shear-rate range and were confined mainly to the low-shear region with the cone–plate geometries. To ensure an identical shear history for all geometries, only the ascending flow curves were used for subsequent rheological model fitting and data analysis.

### 2.3. Rheological Modeling

The experimentally obtained flow curves were fitted using four common rheological models: Newtonian, Power law (Ostwald de Waele), Herschel–Bulkley, and Casson. Model fits were compared descriptively using the software-reported correlation coefficient (*r*) and Chi^2^, together with the fitted yield-stress parameter (*τ*_0_), where applicable (Table S2). The Power law model was retained as a common empirical framework for comparing the measuring geometries within the investigated shear-rate range, based on its high *r* values and consistently interpretable parameters *K* and *n*.

The Power law model is defined by the following equation:

(1) $$\tau =K{\dot{\gamma }}^{n}$$

where τ (Pa) represents shear stress, $\dot{\gamma }$ (s^−1^) is the shear rate, *K* (Pa·s^n^) is the consistency index, and *n* (dimensionless) is the flow behavior index. For each ascending flow curve, the values of *K*, *n*, and the correlation coefficient (*r*) were calculated and used for comparisons of the three measuring geometries. The correlation coefficient, *r*, was used as an indicator of the goodness-of-fit of the applied rheological model.

To estimate the effect of omitting a non-Newtonian correction, a sensitivity analysis was performed using the exact Power law Couette solution described by Skadsem and Saasen [14]. The solution was evaluated separately for both CC27 DG annuli using the geometry dimensions and the individual flow indices (*n* = 0.7305–0.7964), and the rotor torques were summed. The ratio of the consistency inferred using the factory conversions to the specified model consistency was normalized to unity at *n* = 1. Relative changes in *K*, the equivalent shear rate, and local wall shear rates were calculated under steady laminar, homogeneous, no-slip conditions (Table S3).

### 2.4. Statistical Analysis

Statistical analysis was performed using OriginPro 2019 software (v. 9.6.5.169; OriginLab, Northampton, MA, USA). Measurements were carried out in duplicate for each blood sample and each measuring geometry, and data are expressed as mean ± standard deviation (SD).

Statistical significance of differences in the consistency index (*K*), flow behavior index (*n*), and correlation coefficient (*r*) values among the three measuring geometries was determined using the Friedman ANOVA test for related samples. When the Friedman test indicated a statistically significant overall difference, pairwise comparisons were performed using the Wilcoxon signed-rank test with Holm adjustment for multiple comparisons. The effect size of the Friedman test was expressed as Kendall’s coefficient of concordance, *W* = χ^2^/[*n*(*k* − 1)]. Statistical significance was set at *p* < 0.05.

## 3. Results

Whole blood flow curves were obtained from five donors using each of the three measuring geometries, and the corresponding Power law parameters were subsequently calculated. The consistency index (*K*), flow behavior index (*n*), and correlation coefficient (*r*) obtained for each donor and measuring geometry are summarized in Table 1. Descriptively, the mean consistency index (*K*) was lowest for CC27 DG (0.0181 ± 0.0039 Pa·s^n^), whereas higher values were obtained with the cone–plate geometries, reaching 0.0239 ± 0.0055 Pa·s^n^ for C50 1° and 0.0242 ± 0.0059 Pa·s^n^ for C35 2°. The mean flow behavior index (*n*) was highest for CC27 DG (0.7712 ± 0.0220), followed by C35 2° (0.7384 ± 0.0105) and C50 1° (0.7270 ± 0.0250). Correlation coefficients were high for all three geometries, with the highest mean value obtained for CC27 DG (0.9991 ± 0.0006), followed by C50 1° (0.9960 ± 0.0040) and C35 2° (0.9949 ± 0.0028).

The Power law model yielded high *r* values across all geometries, numerically comparable to those of the Casson and Herschel–Bulkley fits, whereas the Newtonian model showed lower *r* values. The yield-stress models gave small *τ*_0_ values, ranging from 0.017 to 0.036 Pa (Table S2). The Newtonian-normalized sensitivity analysis estimated an overestimation of *K* by only 0.38–0.51% (mean 0.43%), corresponding to an equivalent shear-rate increase of 0.48–0.69% (mean 0.56%). The flow index *n* and correlation coefficient *r* remained unchanged because the adjustment was constant for each Power law curve (Table S3).

**Figure 1:**
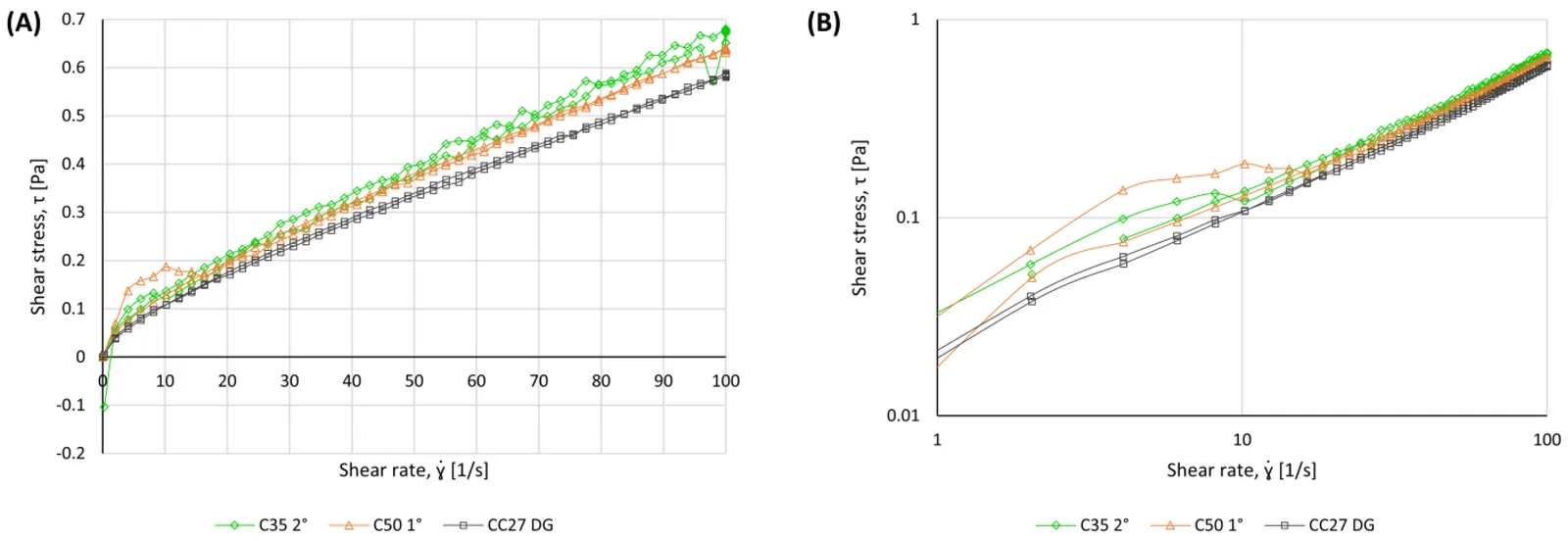
Representative whole-blood flow curves measured using double-gap coaxial cylinder (CC27 DG), 50 mm/1° cone-plate (C50 1°), and 35 mm/2° cone-plate (C35 2°) geometries, presented on linear (**A**) and log–log (**B**) scales. Data shown correspond to Donor 1; both the ascending and descending branches are shown.

The representative whole blood flow curves obtained for Donor 1 using the three measuring geometries are shown in Figure 1. The linear representation (Figure 1A) highlights differences in measured shear stress and point-to-point variability, while the log–log representation (Figure 1B) provides a clearer view of the lower shear rate region. For all geometries, shear stress increased with increasing shear rate over the investigated range (0–100 s^−1^). The curves showed generally similar profiles, although differences in measured shear stress were observed across the shear-rate range. At higher shear rates, the CC27 DG curve was positioned below those obtained with the two cone–plate geometries, while the highest shear stress values were observed with C35 2°. In addition, at higher shear rates, the smaller cone geometry (C35 2°) showed greater point-to-point variability in shear stress, with more pronounced fluctuations along the flow curve. Occasional negative values were also observed in individual C35 2° measurements, generally at the final point of the descending branch. In contrast, the curves obtained with C50 1° and CC27 DG were smoother, with shear stress values showing a more uniform progression across the higher shear-rate range. The log–log representation further highlights the smoother response and closer agreement between the ascending and descending branches obtained with CC27 DG, while both cone–plate geometries showed more pronounced differences between the two branches in the lower shear rate region. Furthermore, an isolated negative shear stress value recorded for C35 2° at the lowest shear rate of the descending branch could not be displayed on logarithmic axes.

**Figure 2:**
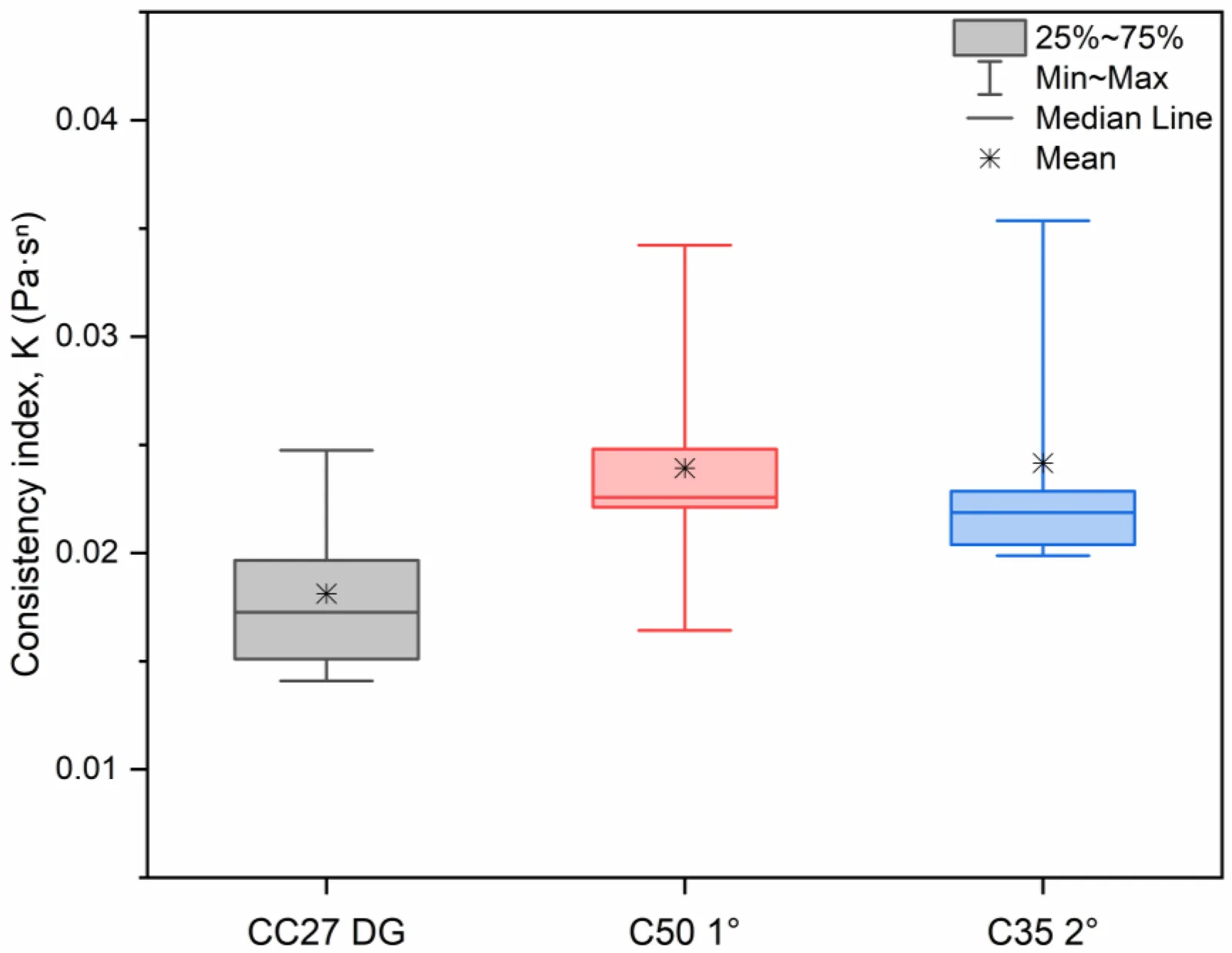
Distribution of the consistency index (*K*) obtained from Power law fitting of whole blood flow curves measured using a double-gap coaxial cylinder (CC27 DG), a 50 mm/1° cone-plate (C50 1°), and a 35 mm/2° cone-plate (C35 2°) geometry. Box plots summarize all ten individual measurements per geometry.

The distribution of the consistency index is shown in Figure 2. *K* values obtained with CC27 DG were lower than those obtained with either cone–plate geometry for all five donors, whereas C50 1° and C35 2° showed similar distributions and overall mean values. A statistically significant overall difference in *K* was detected among the three geometries (Friedman χ^2^(2) = 7.60, *p* = 0.022; Kendall’s *W* = 0.76). The direction of the difference between CC27 DG and each cone–plate geometry was the same in all five donors. However, none of the pairwise comparisons reached statistical significance after Holm adjustment (CC27 DG vs. C50 1°, adjusted *p* = 0.188; CC27 DG vs. C35 2°, adjusted *p* = 0.188; C50 1° vs. C35 2°, adjusted *p* = 0.625).

**Figure 3:**
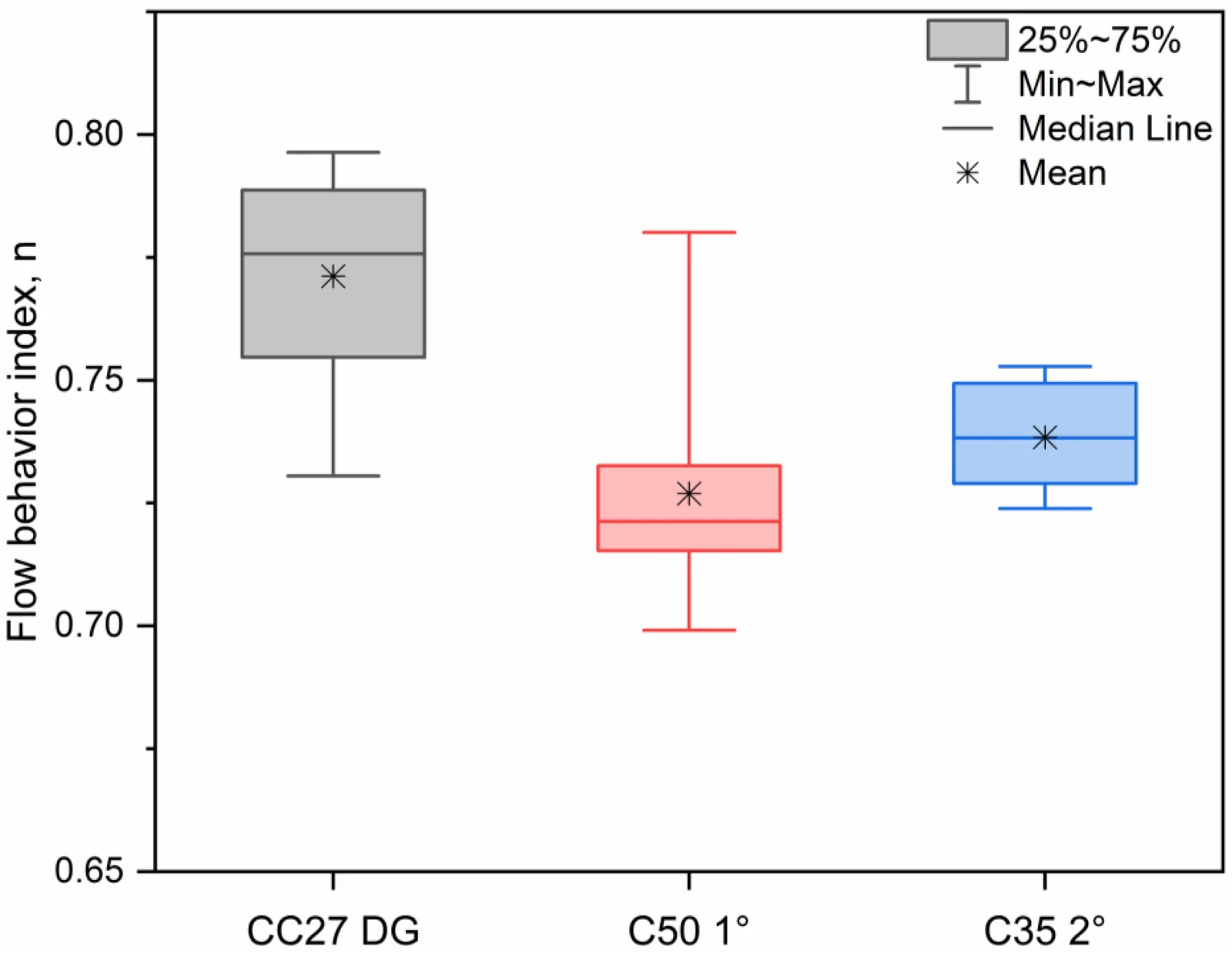
Distribution of the flow behavior index (*n*) obtained from Power law fitting of whole blood flow curves measured using a double-gap coaxial cylinder (CC27 DG), a 50 mm/1° cone-plate (C50 1°), and a 35 mm/2° cone-plate (C35 2°) geometry. Box plots summarize all ten individual measurements per geometry.

Figure 3 shows the distribution of the flow behavior index. All *n* values were in a range of approximately 0.7–0.8, indicating non-Newtonian, shear-thinning behavior of whole blood. CC27 DG showed the highest mean *n* value, C50 1° the lowest, and C35 2° an intermediate value. However, no statistically significant overall difference in *n* was observed among the three geometries (Friedman χ^2^(2) = 2.80, *p* = 0.247; Kendall’s *W* = 0.28).

**Figure 4:**
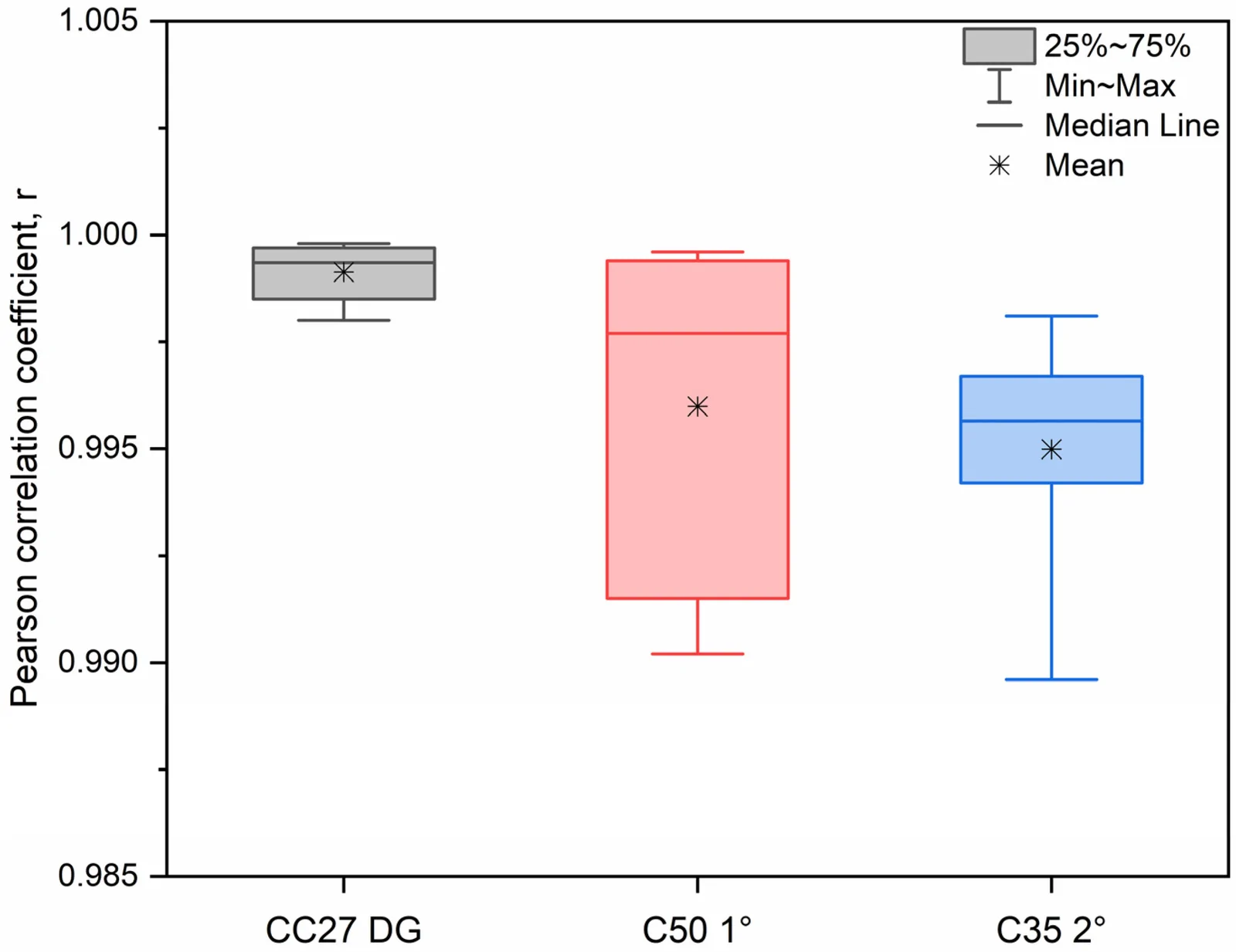
Distribution of the correlation coefficient (*r*) describing the quality of Power law model fitting for whole blood flow curves measured using a double-gap coaxial cylinder (CC27 DG), a 50 mm/1° cone-plate (C50 1°), and a 35 mm/2° cone-plate (C35 2°) geometry. Box plots summarize all ten individual measurements per geometry.

The distribution of correlation coefficients obtained from Power law fitting, indicating the goodness-of-fit of the applied rheological model, is presented in Figure 4. While all measurements resulted in high *r* values (≥0.989), CC27 DG consistently yielded the highest *r* values across the five donors, with values closely approaching 1.000. Cone geometries C50 1° and C35 2° showed slightly lower goodness-of-fit values. A statistically significant overall difference in *r* was observed among the three geometries (Friedman χ^2^(2) = 8.40, *p* = 0.015; Kendall’s *W* = 0.84). Pairwise Wilcoxon signed-rank tests did not identify statistically significant differences after Holm adjustment (CC27 DG vs. C50 1°, adjusted *p* = 0.188; CC27 DG vs. C35 2°, adjusted *p* = 0.188; C50 1° vs. C35 2°, adjusted *p* = 0.313).

## 4. Discussion

The present study evaluated the influence of the geometry of the rotational element on steady-shear rheological characterization of whole blood under controlled experimental conditions. All three geometries identified shear-thinning behavior. The Friedman tests provided evidence of significant overall geometry effects on *K* and *r*, whereas no significant overall effect was detected for *n*. However, no Holm-adjusted pairwise comparison reached statistical significance, so the omnibus results do not identify which specific geometries differ. The lower mean *K* and higher mean *r* observed with CC27 DG therefore represent descriptive patterns rather than statistically confirmed pairwise differences. With only five donors, the pairwise tests had limited power; their non-significant results should not be interpreted as evidence of equivalence between geometries.

**Figure 5:**
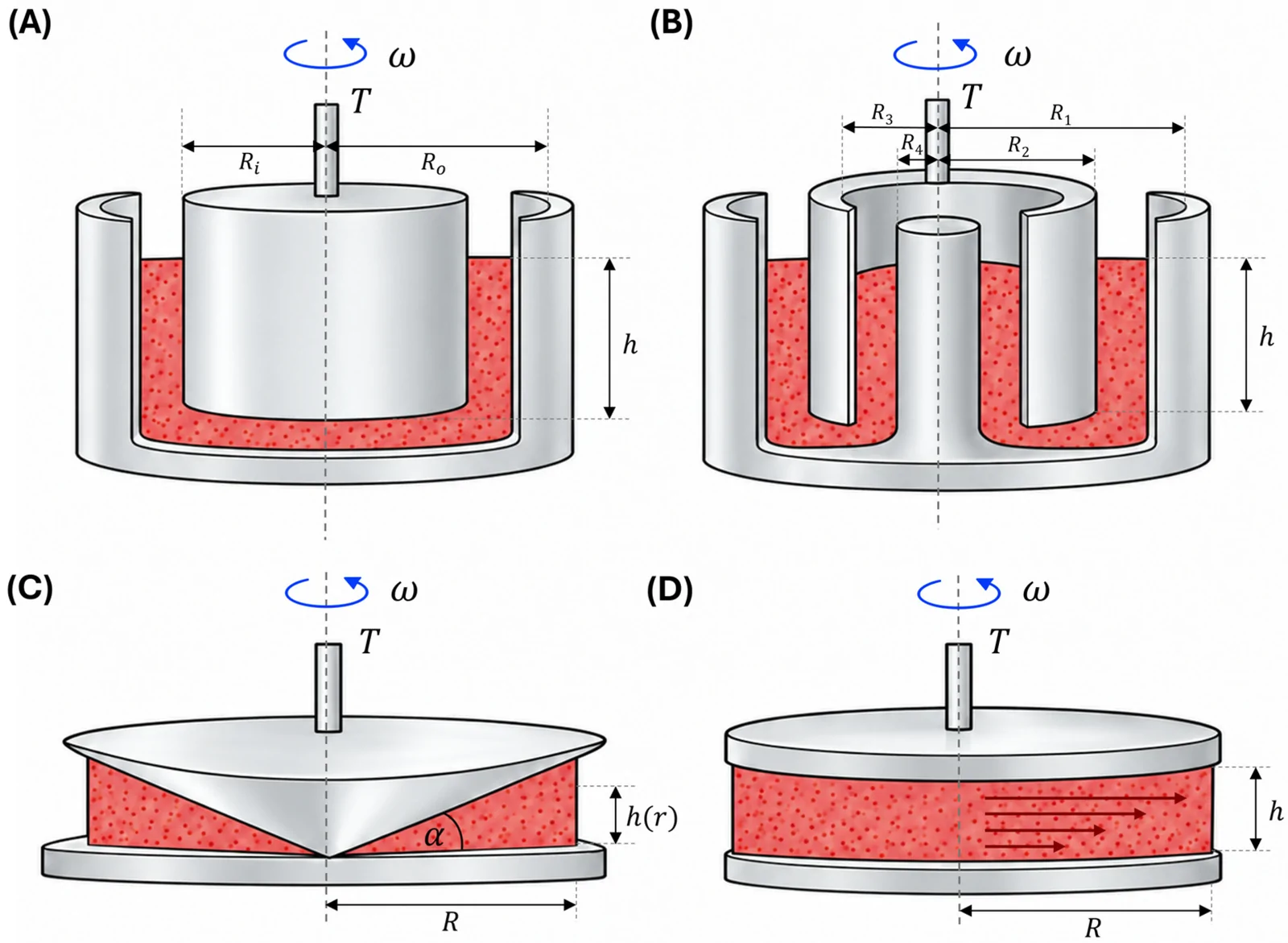
Schematic overview of rotational rheometer geometries and the fundamental measurement principles of conventional concentric cylinder (**A**), double-gap concentric cylinder (**B**), cone–plate (**C**), and parallel plate (**D**) systems. Rotational rheometers directly record torque (*T*) and angular velocity (ω), whereas shear rate ($\dot{\gamma }$) and shear stress (τ) are calculated using geometry-specific transformation equations. In the conventional concentric cylinder system (**A**), these relationships depend on the inner and outer cylinder radii (*R_i_* and *R_o_*), the annular gap (*R_o_* − *R_i_*), and the immersed height (*h*). In the double-gap system (**B**), a hollow cylindrical rotor shears the sample within two annular gaps. The outer gap is defined as *R*_1_ − *R*_2_, where *R*_1_ is the inner radius of the outer stationary cylinder and *R*_2_ is the outer radius of the rotor. The inner gap is defined as *R*_3_ − *R*_4_, where *R*_3_ is the inner radius of the rotor and *R*_4_ is the outer radius of the central stationary cylinder. Both rotor surfaces contribute to the measured torque over the immersed height (*h*). In the cone–plate system (**C**), the gap increases proportionally with radial distance (*r*) and is determined by the cone angle (α), resulting in an approximately uniform shear rate for small cone angles. In the parallel-plate system (**D**), the gap is constant across the sample, while the shear rate increases with radial distance (*r*) from the rotational axis, resulting in a radially non-uniform shear field. *R* denotes the cone or plate radius.

These geometry-dependent differences first need to be considered in the context of the fundamental principles of rotational rheometry. Namely, rheometers do not directly measure shear stress (τ) and shear rate ($\dot{\gamma }$), but torque (*T*) and rotational velocity (ω), which are then converted into rheological parameters using geometry-specific transformation equations [15,16]. This has also been emphasized in hemorheological guidelines, which state that the relationship between measured torque, rotational speed, shear rate, and shear stress depends on the geometry of the rotational element [16]. Therefore, differences in measuring geometry impact not only the generated flow field but also the mathematical relationship between the directly measured and the calculated rheological variables. The most commonly used geometries in rotational rheometry (conventional concentric-cylinder, double-gap concentric-cylinder, cone–plate and parallel-plate geometry) and their fundamental measurement principles are illustrated in Figure 5.

For a conventional concentric-cylinder system, the shear stress at a radial position *r* can be expressed as [15]:

(2) $$\tau \left(r\right)=\frac{T}{2\pi r^{2}h}$$

where *h* is the height of the immersed cylinder. For sufficiently small annular gaps, the shear rate can be approximated as [15]:

(3) $$\dot{\gamma }\approx \frac{\omega }{\frac{R_{o}}{R_{i}}-1}=\frac{\omega R_{i}}{R_{o}-R_{i}}$$

where *R_i_* and *R_o_* are the inner and outer cylinder radii, respectively. With increasing gap width, shear-rate determination becomes more dependent on the velocity profile and rheological properties of the investigated material. This expression illustrates the conventional single-gap approximation; the CC27 DG data in the present study were calculated using the factory conversion factors specified in Section 2.2.

In cone–plate systems, the gap increases proportionally with radial distance: *h*(*r*) = *r* tan *α*, and given that the circumferential velocity also increases proportionally: *v*(*r*) = *rω*, the shear rate remains approximately constant for small cone angles [15]:

(4) $$\dot{\gamma }=\frac{\omega }{\tan\alpha }\approx \frac{\omega }{\alpha }$$

while shear stress is given by:

(5) $$\tau =\frac{3T}{2\pi R^{3}}$$

where *R* is the cone radius and α is the cone angle expressed in radians. This near-uniform shear field represents one of the main theoretical advantages of cone–plate geometry, whereas conventional concentric-cylinder systems are characterized by radial, geometry- and gap-dependent stress and shear-rate distributions [15]. Therefore, even when sample handling, temperature, and the imposed shear protocol are identical, the rheological response derived from the instrument signal can be influenced by the selected measuring geometry.

In the observed data, the double-gap concentric-cylinder system showed the highest mean *r* and the smoothest flow curves; these observations describe its performance under the present protocol and do not establish statistically significant pairwise superiority. This result was expected considering the general suitability of concentric-cylinder systems for low-viscosity fluids. Their configuration confines the sample and prevents material to flow out of the measuring gap, while the relatively large contact surface improves the response of fluids producing low torque signals. A recent whole-blood study using a concentric-cylinder rheometer described this geometry as a conventional choice for low-viscosity samples because of its large contact surface and limited evaporation [17]. Hemorheological guidelines also highlight that instrument sensitivity is particularly important when measuring blood at low shear and that the measuring system needs to provide sufficient sensitivity and accuracy for the relatively weak rheological response of blood [16].

The double-gap configuration introduces a hollow rotor and two annular shearing regions, both contributing to the measured torque. Malik et al. reported a broader measurable viscosity range with a double concentric-cylinder system than with conventional coaxial-cylinder and cone–plate systems [18]. Scorza et al. likewise emphasized the suitability of double-interstice geometries for very low viscosity liquids, while noting that instrumental and mechanical effects become proportionally more important as viscosity decreases [19]. These properties may help explain the smoother curves and numerically higher mean *r* observed with CC27 DG, but do not establish pairwise superiority or greater absolute accuracy.

The absence of a non-Newtonian correction in the CC27 DG shear-rate calculation should also be considered. Both annuli have outer-to-inner radius ratios of approximately 1.022. The Newtonian-normalized Power law analysis estimated only a 0.38–0.51% overestimation of *K*, with no change in *n* or *r* (Table S3). This effect is much smaller than the approximately 24–25% lower mean *K* obtained with CC27 DG compared with the cone–plate geometries. Under the model assumptions, accounting for this effect would slightly decrease CC27 DG *K*, rather than explain its lower value. It should be noted that these estimates represent a theoretical sensitivity analysis and were not experimentally validated.

Cone–plate geometries have different advantages, with the approximately homogeneous shear field and a nearly constant shear rate across the sample for small cone angles being the most important ones [15,20]. These properties, together with requirements for relatively low sample volumes, may explain their widespread use in blood rheometry, and previous studies on hemorheology have successfully employed cone–plate systems with different cone diameters and angles [21]. In the present study, the two cone–plate geometries differed in their analytical performance, with the C50 1° geometry producing smoother flow curves than C35 2° (especially at higher shear rates), and better overall goodness-of-fit. The cone radius is a possible contributing factor for such results since the torque corresponding to a given shear stress is proportional to *R*^3^ in an ideal system; accordingly, the 25 mm radius of C50 cone compared with the 17.5 mm radius of C35 corresponds to a theoretical torque ratio of around 3 [(25/17.5)^3^ ≈ 2.9].

Therefore, the larger cone provides a stronger measurable torque signal, which is particularly relevant at higher shear rates when blood viscosity is decreased. For this reason, larger cone radii are generally preferred for low-viscosity suspensions because of the larger shearing area [15]. However, since C50 and C35 differed simultaneously also in the cone angle, the individual contributions of cone diameter and angle cannot be separated in the present study. Reconstruction using *T* = τ/*A* for the representative Donor 1 ascending sweep gave positive torque ranges of approximately 0.038–42.4 μN·m for CC27 DG, 0.012–21.0 μN·m for C50 1°, and 0.015–7.59 μN·m for C35 2°. These illustrative magnitudes support consideration of geometry-dependent torque response, but do not independently establish signal-to-noise ratio or absolute measurement accuracy.

Several other measurement phenomena, including wall slip, gap-setting errors, cell migration or sedimentation, edge effects, secondary flows, may also lead to the geometry-related differences in results. Such effects should be considered as potential sources of variability rather than direct explanations for these differences. For example, studies of complex fluids have attributed discrepancies between plate-type geometries to wall slip and inaccurate gap setting [20], while experiments with heterogeneous emulsions have demonstrated that phase segregation may affect cone–plate measurements differently from coaxial-cylinder measurements [22]. These considerations are also directly relevant to blood since hemorheological guidelines indicate that sedimentation of RBCs at low shear can produce a cell-poor, lower viscosity layer in cone–plate systems and consequently reduce the measured torque and apparent viscosity [16], while hemorheological experimental studies have similarly shown that aggregation, sedimentation, measurement duration, and geometry can interact to affect the steady-shear results [21]. Furthermore, studies of highly viscous materials also demonstrate geometry-specific limitations such as edge fracture and flow instabilities, further illustrating that no single plate-based rheological configuration is optimal across a wide viscosity range [23].

Despite these geometry-dependent differences, all three measuring systems in this study consistently characterized whole blood as a shear-thinning fluid, with mean *n* values remaining within a relatively narrow range (approx. 0.7–0.8) with no statistically significant overall effect of geometry on this parameter. In contrast, the consistency index *K* and the goodness-of-fit parameter *r* showed significant overall geometry effects. These findings preserve the qualitative classification of whole blood as shear-thinning while supporting consideration of measuring geometry when interpreting quantitative fitted parameters; they do not establish statistically significant differences between specific geometry pairs. A similar conclusion was reached by Alves et al., who demonstrated that whole blood rheological data and fitted constitutive-model parameters were significantly influenced by the measuring geometry, although the Power law model showed comparatively better agreement among different measuring systems in comparison to several other models. Their reported Power law parameters also varied among cone–plate and parallel-plate configurations, emphasizing the need to interpret fitted coefficients in relation to the experimental geometry of the rotational element [21].

The interpretation of *K* and *n* should also take into account the shear-rate range over which the model is fitted. Marinakis et al. demonstrated that Power law coefficients derived from blood rheology measurements at low shear rates did not reliably predict viscosity at higher shear rates, with deviations occurring above 5 s^−1^ in their experimental system [24]. Therefore, Power law coefficients should be considered as empirical descriptors of blood behavior over a specific experimental range rather than universal intrinsic constants. In the present study, the same protocol and fitting range were applied to all three geometries, which strengthens the interpretation that the observed systematic shifts in *K* primarily reflect differences associated with the measuring geometries. The Power law model does not represent an apparent yield stress or the time-dependent and elastic contributions associated with erythrocyte aggregation. Its use here is therefore restricted to an empirical comparison of measuring geometries over the investigated shear-rate range and should not be extrapolated to vanishing shear rates. More comprehensive thixotropic elasto-viscoplastic (TEVP) formulations account for structural evolution, viscoelasticity, and yielding [25,26]. The present protocol was not designed to independently characterize these contributions or reliably identify the corresponding model parameters.

The geometry dependence needs to be additionally considered in the context of the strongly shear-dependent microstructure of blood. Whole blood viscosity reflects the combined contributions of hematocrit, plasma viscosity, erythrocyte aggregation, and erythrocyte deformability, with aggregation being particularly important at low shear rates and deformability at higher shear rates [27]. Vitkova et al. demonstrated that transitions in erythrocyte microscopic dynamics, including tank-treading and tumbling, can significantly alter the macroscopic viscosity of RBC suspensions [28]. However, it should be noted that those experiments were performed in dilute RBC suspensions with hematocrit below 10% and with plasma proteins removed to inhibit rouleaux formation, and therefore should not be directly extrapolated quantitatively to whole blood at physiological hematocrit. The cellular microstructure of blood changes during flow due to erythrocyte aggregation and disaggregation, deformation, and orientation [6]. Although the same nominal shear rate conditions were applied to all measuring geometries, differences in the spatial distribution of shear and local flow conditions may influence the blood structural evolution. Geometry-dependent restructuring may therefore contribute to the observed rheological differences, although this contribution could not be directly assessed in the present study. These findings support the view that rheological parameters obtained from whole blood flow curves represent an integrated response determined by blood microstructure, the imposed shear conditions, the selected measuring geometry, and the mathematical model used for data analysis.

From a practical perspective, the results of this study indicate that the selection of measuring geometry for blood rheometry has to involve a balance between analytical performance and sample availability. Table 2 summarizes the observed curve characteristics and sample-volume requirements of the three systems. Minimizing blood volume is an important methodological consideration, particularly for repeated measurements or limited sample availability [29]. The observed patterns suggest potential practical advantages of CC27 DG and C50 1°, but do not establish a statistically confirmed ranking. The use of cone–plate systems in routine applications is also supported by reported performance of automated blood viscometers [30].

The significant overall geometry effects observed in the present study reinforce the need for more specific methodological standardization in hemorheological research, although the existing hemorheological guidelines already emphasize strict control of analytical conditions, including measurement temperature, shear conditions, sample handling, and instrument performance [16]. Nevertheless, a systematic review of blood viscosity methodologies identified substantial heterogeneity among published studies in sample volume, shear-rate range, equipment, storage and measurement conditions, and it was concluded that greater methodological uniformity is necessary for the clinical applicability and comparability of hemorheological measurements [29]. The present findings suggest that the measuring geometry itself should be considered as an additional component of such standardization, and future studies should explicitly report the geometry type and dimensions, sample volume, temperature, shear-rate range and shear history, time between blood collection and measurement, as well as the mathematical model and fitting range used for data analysis. Such reporting would improve reproducibility and enable distinguishing the true biological differences from variability introduced by the measurement system.

Several limitations should be considered when interpreting the results of the study. First, the study included only five independent donors, limiting statistical power, particularly for pairwise comparisons after adjustment for multiple testing. With five donors, the smallest attainable two-sided exact Wilcoxon signed-rank *p*-value is 0.0625 (0.1875 after Holm adjustment for three comparisons), so no pairwise comparison could reach *p* < 0.05 regardless of the magnitude of the difference. Consequently, the significant omnibus findings support an overall geometry effect, while geometry-specific rankings remain descriptive and require confirmation in larger studies. Second, although three commonly used measuring geometries were tested, several alternative geometries are available, and the present comparison was performed using a single rheometer and measurement protocol. Moreover, the two cone–plate geometries differed simultaneously in diameter and cone angle, so the individual contribution of these geometric factors could not be determined. Matched comparisons using C35 1° or C50 2° geometries would help separate these effects; however, these geometries were not available in the present study. Measurements at 25 °C do not provide physiological reference values, and whether the geometry-dependent patterns persist at 37 °C requires separate investigation. Eventually, although differences in curve smoothness and model fit may be related to geometry-dependent torque sensitivity, torque signal quality was not independently quantified in the present study. Further studies involving larger and more diverse donor populations, additional geometries, and systematic variation in individual geometric parameters would thus strengthen the generalizability of these findings.

## 5. Conclusions

Under the standardized conditions of this study, measuring geometry showed significant overall effects on the consistency index *K* and Power law goodness-of-fit *r*, while no significant overall effect was detected for *n*. All geometries identified shear-thinning behavior. No Holm-adjusted pairwise comparison reached statistical significance; therefore, the lower mean *K*, higher mean *r*, and smoother curves observed with CC27 DG should be interpreted descriptively. C50 1° also produced smooth curves with a smaller sample volume requirement. These findings support explicit reporting and standardization of measuring geometry, while larger studies are needed to confirm specific differences between geometries.

## Figures and Tables

**Table 1:** Consistency index (*K*), flow behavior index (*n*), and goodness-of-fit (*r*) obtained from Power law model fitting of whole blood flow curves measured using three rheometer geometries.

Parameter	Measurement Geometry	Donors					Mean ± SD *
		1	2	3	4	5	
*K* [Pa·s^n^]	CC27 DG	0.0173 ± 0.0010	0.0147 ± 0.0006	0.0192 ± 0.0006	0.0245 ± 0.0003	0.0149 ± 0.0011	0.0181 ± 0.0039
	C50 1°	0.0235 ± 0.0019	0.0204 ± 0.0025	0.0199 ± 0.0049	0.0334 ± 0.0012	0.0226 ± 0.0004	0.0239 ± 0.0055
	C35 2°	0.0222 ± 0.0010	0.0224 ± 0.0006	0.0200 ± 0.0001	0.0352 ± 0.0002	0.0211 ± 0.0010	0.0242 ± 0.0059
*n*	CC27 DG	0.7623 ± 0.0107	0.7925 ± 0.0054	0.7376 ± 0.0100	0.7760 ± 0.0047	0.7877 ± 0.0124	0.7712 ± 0.0220
	C50 1°	0.7137 ± 0.0206	0.7362 ± 0.0295	0.7398 ± 0.0571	0.7256 ± 0.0099	0.7197 ± 0.0060	0.7270 ± 0.0250
	C35 2°	0.7367 ± 0.0033	0.7333 ± 0.0060	0.7503 ± 0.0013	0.7247 ± 0.0011	0.7470 ± 0.0082	0.7384 ± 0.0105
*r*	CC27 DG	0.9995 ± 0.0004	0.9991 ± 0.0009	0.9988 ± 0.0011	0.9990 ± 0.0007	0.9994 ± 0.0006	0.9991 ± 0.0006
	C50 1°	0.9950 ± 0.0064	0.9955 ± 0.0056	0.9949 ± 0.0066	0.9978 ± 0.0017	0.9969 ± 0.0028	0.9960 ± 0.0040
	C35 2°	0.9956 ± 0.0003	0.9929 ± 0.0046	0.9949 ± 0.0009	0.9973 ± 0.0008	0.9944 ± 0.0052	0.9949 ± 0.0028

* Mean ± SD of all ten individual measurements.

**Table 2:** Practical considerations for the rotational geometries evaluated in the present study.

Geometry	Description	Approx. Sample Volume	Performance Observed in the Present Study *	Main Practical Advantage	Main Consideration
CC27 DG	Double-gap concentric-cylinder system with two annular shearing regions	3.0 mL	Highest observed mean goodness-of-fit; smoothest flow curves	Observed smooth flow curves and suitability for low-viscosity measurements	Largest sample-volume requirement
C50 1°	Large-radius cone–plate; approximately uniform shear rate	1.0 mL	Good fit and smooth flow response; intermediate between DG and C35	Favorable balance between measurement performance and sample economy	Mean *K* approximately 32% higher than CC27 DG; greater volume than C35
C35 2°	Smaller-radius cone–plate; approximately uniform shear rate	0.4 mL	Lowest mean goodness-of-fit and greater high-shear scatter	Minimal sample requirement	Smaller torque magnitude in the representative sweeps; greater variability

* Performance descriptions summarize observations under the present protocol and do not indicate statistically confirmed pairwise superiority.

## Data Availability

The data supporting the findings of this study are included within the article. Additional anonymized data are available from the corresponding author upon reasonable request, subject to ethical approval and participant confidentiality.

## Supplementary Materials

The following supporting information can be downloaded at https://www.mdpi.com/article/10.3390/mps9050150/s1, Table S1: Characteristics of the three measuring geometries used for whole blood rheometry; Table S2: Comparison of rheological model fits for whole blood measured using three rheometer geometries; Table S3: Power law sensitivity analysis for the CC27 DG measuring geometry.
