# Supplementary material for: Geometry-Dependent Variability in Rotational Rheometry of Whole Blood: Implications for Flow Curve Measurement and Protocol Standardization
Source: Methods Protoc. 2026 Oct 14;9(5):150. doi: 10.3390/mps9050150 (PMC13707631; doi:10.3390/mps9050150)
Supplement: Supplementary file 1 [file mps-09-00150-s001.zip › mps-4565758-supplementary.pdf]

Table S1. Characteristics of the three measuring geometries used for whole blood rheometry.

**Characteristic**	**CC27 DG/Ti**	**C50 1°/Ti**	**C35 2°/Ti**
Description	Double-gap cylinder	Cone–plate	Cone–plate
Geometry [*]	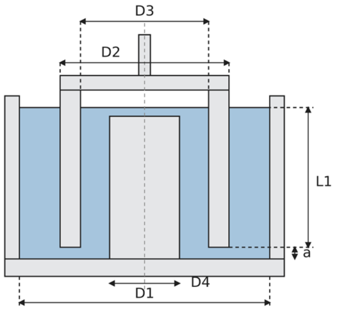 D1: outer cup inner diameter; D2: rotor outer diameter; D3: rotor inner diameter; D4: central stationary cylinder outer diameter. L1: immersed rotor length; a: axial clearance at the rotor bottom.	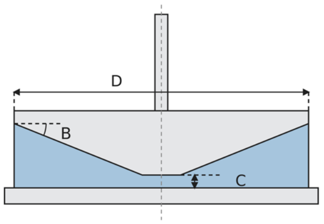 D: cone diameter; B: cone angle; C: cone truncation distance.	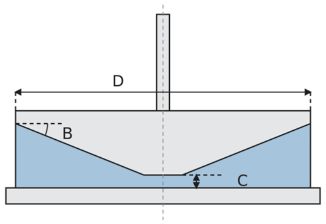 D: cone diameter; B: cone angle; C: cone truncation distance.
Order number	222-2099	222-2456	222-2113
Serial number	02240564	01241418	02200284
Material designation	Ti	Ti	Ti
Cone diameter D (mm)	—	49.998	34.9990
Cone angle B (°)	—	1	1.9900
Truncation C (mm)	—	0.052	0.0980
D1 (mm)	27.20	—	—
D2 (mm)	26.6000	—	—
D3 (mm)	21.2760	—	—
D4 (mm)	20.81	—	—
Immersed length L1 (mm)	40.01	—	—
Axial distance a (mm)	4.0	—	—
Outer radial gap (mm) [**]	0.300	—	—
Inner radial gap (mm) [**]	0.233	—	—
Mass inertia I ($kg·m^2$)	6.766×10^{-6}	6.42×10^{-6}	1.78×10^{-6}
Compliance J (rad/(N·m))	0.00000	0.003	0.0030
Sample volume V (mL)	3.0	1	0.40
A-factor (Pa/(N·m)) [***]	13,720	30,560	89,100
M-factor ((s^{-1})/(rad·s^{-1})) [***]	45.810	57.3	28.790

[*] Diagrams reproduce the dimension labels based on the certificate schematics; they are not drawn to scale; the cone angle and the radial and axial gaps are enlarged for clarity.

[**] Derived from the reported diameters: outer radial gap = (D1 − D2)/2; inner radial gap = (D3 − D4)/2.

[***] Factor A converts torque T to shear stress ($\tau = AT$), and M converts angular velocity ω to shear rate ($\dot{\gamma} = M\omega$).

Table S2. Comparison of rheological model fits for whole blood measured using three rheometer geometries.

Geometry	Model	Correlation coefficient, *r*	Chi²	Yield stress, τ_0 (Pa)
CC27 DG	Newtonian	0.9816 ± 0.0045	0.0566 ± 0.0254	N/A
	Power law	0.9991 ± 0.0006	0.0028 ± 0.0025	N/A
	Herschel–Bulkley	0.9994 ± 0.0005	0.0019 ± 0.0012	0.0177 ± 0.0122
	Casson	0.9996 ± 0.0001	0.0011 ± 0.0004	0.0168 ± 0.0044
C50 1°	Newtonian	0.9687 ± 0.0093	0.1060 ± 0.0495	N/A
	Power law	0.9960 ± 0.0040	0.0121 ± 0.0103	N/A
	Herschel–Bulkley	0.9975 ± 0.0025	0.0074 ± 0.0063	0.0362 ± 0.0263
	Casson	0.9977 ± 0.0020	0.0067 ± 0.0048	0.0266 ± 0.0076
C35 2°	Newtonian	0.9713 ± 0.0026	0.1160 ± 0.0539	N/A
	Power law	0.9949 ± 0.0028	0.0186 ± 0.0089	N/A
	Herschel–Bulkley	0.9957 ± 0.0028	0.0154 ± 0.0087	0.0210 ± 0.0101
	Casson	0.9953 ± 0.0034	0.0174 ± 0.0110	0.0247 ± 0.0073

Values are mean ± SD of ten individual fits per geometry and model, except for Herschel–Bulkley, for which physically inadmissible solutions with negative *K* or *n* were excluded. The retained fit counts were 8 for CC27 DG, 10 for C50 1°, and 5 for C35 2°. N/A – not applicable.

Table S3. Power law sensitivity analysis for the CC27 DG measuring geometry.

Donor	Measurement	*n*	*K* bias (%)	Shear-rate bias (%)	Wall shear-rate deviation, smaller-radius wall (%)	Wall shear-rate deviation, larger-radius wall (%)
1	1	0.7698	+0.43	+0.56	+0.66	-0.67
	2	0.7547	+0.46	+0.61	+0.72	-0.72
2	1	0.7963	+0.38	+0.48	+0.56	-0.57
	2	0.7887	+0.40	+0.50	+0.59	-0.60
3	1	0.7305	+0.51	+0.69	+0.81	-0.82
	2	0.7446	+0.48	+0.64	+0.76	-0.76
4	1	0.7726	+0.43	+0.55	+0.65	-0.66
	2	0.7793	+0.41	+0.53	+0.62	-0.63
5	1	0.7964	+0.38	+0.48	+0.56	-0.57
	2	0.7789	+0.42	+0.53	+0.63	-0.63

K bias denotes the relative overestimation of consistency in the Newtonian-normalized model comparison. The shear-rate bias denotes the equivalent increase relative to the nominal shear rate at the same stress. Wall shear-rate deviations refer to the inner annulus and are calculated relative to the Newtonian solution at the same angular velocity.

Geometry: the inner annulus is bounded by radii 10.405 and 10.638 mm; its rotating wall is at the larger radius. The outer annulus is bounded by radii 13.300 and 13.600 mm; its rotating wall is at the smaller radius. Rotor length L = 40.01 mm. Conversion factors: A = 13,720 Pa/(N·m) and M = 45.810 (s^{-1})/(rad·s^{-1}). *n* = Power law flow behavior index of each CC27 DG measurement. Assumptions: homogeneous Power law fluid, steady laminar azimuthal flow, no slip, common consistency in both annuli and equal immersed lengths. End effects, yield stress, structural evolution, and secondary flows were not modeled.
